# Ratio of Monocytes to Lymphocytes in Peripheral Blood Identifies Adults at Risk of Incident Tuberculosis Among HIV-Infected Adults Initiating Antiretroviral Therapy

**DOI:** 10.1093/infdis/jit494

**Published:** 2013-09-16

**Authors:** Vivek Naranbhai, Adrian V. S. Hill, Salim S. Abdool Karim, Kogieleum Naidoo, Quarraisha Abdool Karim, George M. Warimwe, Helen McShane, Helen Fletcher

**Affiliations:** 1 Wellcome Trust Centre for Human Genetics; 2 The Jenner Institute, Nuffield Department of Medicine, University of Oxford, United Kingdom; 3 Center for the AIDS Program of Research in South Africa, University of KwaZulu Natal, Durban

**Keywords:** tuberculosis, HIV, combination antiretroviral therapy, monocytes, lymphocytes, ML ratio

## Abstract

***Background.*** Eight decades ago, the ratio of monocytes to lymphocytes (hereafter, the “ML ratio”) was noted to affect outcomes of mycobacterial infection in rabbits. Recent transcriptomic studies support a role for relative proportions of myeloid and lymphoid transcripts in tuberculosis outcomes. The ML ratio in peripheral blood is known to be governed by hematopoietic stem cells with distinct biases.

***Methods.*** The predictive value of the baseline ML ratio was modeled in 2 prospective cohorts of HIV-infected adults starting cART in South Africa (primary cohort, 1862 participants; replication cohort, 345 participants). Incident tuberculosis was diagnosed with clinical, radiographic, and microbiologic methods per contemporary guidelines. Kaplan-Meier survival analyses and Cox proportional hazards modeling were conducted.

***Results.*** The incidence rate of tuberculosis differed significantly by baseline ML ratio: 32.61 (95% confidence interval [CI], 15.38–61.54), 16.36 (95% CI, 12.39–21.23), and 51.80 (95% CI, 23.10–101.71) per 1000 patient-years for ML ratios of less than the 5th percentile, between the 5th and 95th percentiles, and greater than the 95th percentile, respectively (*P* = .007). Neither monocyte counts nor lymphocyte counts alone were associated with tuberculosis. After adjustment for sex, World Health Organization human immunodeficiency virus disease stage, CD4^+^ T-cell counts, and previous history of tuberculosis, hazards of disease were significantly higher for patients with ML ratios of less than the 5th percentile or greater than the 95th percentile (adjusted hazard ratio, 2.47; 95% CI, 1.39–4.40; *P* = .002).

***Conclusions.*** The ML ratio may be a useful, readily available tool to stratify the risk of tuberculosis and suggests involvement of hematopoietic stem cell bias in tuberculosis pathogenesis.

Tuberculosis is the leading cause of death in patients commencing combination antiretroviral therapy (cART) in sub-Saharan Africa [1–3], yet practical methods to stratify risk in this population are lacking [4]. If individuals at risk for tuberculosis could be accurately identified, they could be targeted for preventive strategies such as chemoprophylaxis. Risk factors for tuberculosis include immunosuppression, diabetes mellitus, smoking, and the presence of latent *Mycobacterium tuberculosis* infection [5]. However, identifying individuals who have latent *M. tuberculosis* infection or a high risk for developing tuberculosis is a major challenge; tuberculin skin tests (TSTs) and the recently developed interferon γ–release assays are poor at predicting which individuals with latent infection will subsequently go on to develop tuberculosis, particularly in human immunodeficiency virus (HIV)–positive populations [6]. The need to identify groups at risk of tuberculosis is highlighted by the fact that isoniazid preventive therapy is most effective among TST-positive patients [7, 8] as opposed TST-negative individuals [9].

Gene expression studies have identified biomarkers of active tuberculosis that provide insight into immune mechanisms of disease and may be useful for improving the diagnosis of tuberculosis [10, 11]. Fletcher et al recently used whole-transcriptome microarrays to identify correlates of risk of tuberculosis up to 2 years before disease developed in infants who had been vaccinated with BCG at birth (Fletcher et al, unpublished data). Relative abundance of myeloid-specific gene transcripts and lymphoid-specific transcripts at 10 weeks of age were associated with subsequent tuberculosis after stratifying for BCG responses. The observation that the relative proportion of monocytes and lymphocytes may be related to tuberculosis susceptibility is reminiscent of older studies. Between 1921 and 1931, Florence Sabin and colleagues found that numbers of monocytes were increased following experimental infection of rabbits with *Mycobacterium bovis* [12]. They and others reported that the ratio of lymphocytes to monocytes in peripheral blood correlated with extent of disease in both rabbits [13] and humans [14], although the numbers studied were small and the strength of the conclusions that could be reached in humans conceded to be modest. Finally, by experimentally reducing monocyte numbers in rabbits with a monocyte antiserum or conversely increasing the number of monocytes with A-3 phosphatide and then challenging animals with *M. bovis*, they demonstrated that reduced or elevated ratios of monocytes to lymphocytes (hereafter, the “ML ratio”) were associated with enhanced lethality of mycobacterial infection [15]. Lymphoid cells are thought to be the major effector cells in tuberculosis immunity and myeloid cells the major host cell for infection. Therefore, the relative abundance may reflect a balance between effector and target cells. Among infants with altered ratios of myeloid transcripts to lymphoid transcripts in the study by Fletcher et al, however, hematopoietic stem cell– and inflammation-associated transcripts were also altered. Therefore, an alternative explanation is that the relative abundance of these cell types could be a marker of hematopoietic parameters associated with tuberculosis. Collectively, these data support the hypothesis that a high or a low ML ratio may be a correlate of risk for tuberculosis. We evaluated this hypothesis in a cohort of HIV-infected South African adults commencing combination antiretroviral therapy (cART).

## METHODS

### Study Design and Setting

Data in this study were derived from several cohort studies or clinical trials [16–19] conducted at the at the Centre for the AIDS Program of Research in South Africa (CAPRISA) AIDS Treatment (CAT) clinic in Vulindlela, rural KwaZulu Natal, or the CAPRISA eThekwini clinic in Durban, KwaZulu Natal. In each study, prospective follow-up with routine clinical assessments, including specific assessment for tuberculosis and routine measurement of monocyte and lymphocyte counts, was performed. The UKZN Biomedical Research Ethic Committee approved the use of the data from each study.

### Participants Clinical Care, Tuberculosis Diagnosis, and Data Sources

For the primary analysis participants were ambulatory, HIV-seropositive, cART-naive adults older than 18 years who enrolled in care and treatment between April 2004 and April 2011 at the CAT clinic in Vulindlela, rural KwaZulu Natal [16]. Care for HIV infection and/or tuberculosis was provided per the South African treatment guidelines of that time [20–22]. Briefly, patients who were clinically evaluated to have WHO stage IV disease or had a CD4^+^ T-cell count <200 cells/µL were eligible for commencement of a cART regimen comprising 2 nucleoside reverse-transcriptase inhibitors and 1 nonnucleoside reverse-transcriptase inhibitor. During this period, the South African guidelines did not support the routine use of isoniazid preventive therapy for patients receiving cART. For this analysis, 1862 individuals who initiated cART and had at least 1 full blood count measurement available before cART were included. At enrollment, all individuals underwent clinical examination, including clinical screening for tuberculosis and other opportunistic infections. Individuals with active tuberculosis at enrollment were excluded. Participants were clinically evaluated at least twice before enrollment, and monthly after cART commencement. During follow-up after cART initiation, individuals with symptoms and/or signs of tuberculosis were assessed and received a diagnosis per South African guidelines, which include clinical examination, sputum microscopy, and/or culture and chest radiography as indicated [21].

The replication cohort was a cohort of cART-naive adults who self-referred for care at the Prince Cyril Zulu Communicable Diseases Centre, a chest clinic that provides care for the eThekwini (Durban) municipality.

HIV-uninfected women enrolled in the CAPRISA004 trial were included for analyses of the stability of the ML ratio [17]. Routine safety monitoring, including full blood count, was performed at screening (0 months) and at 3, 12, and 24 months. Individuals who had signs or symptoms of tuberculosis were excluded.

For analyses of the impact of HIV acquisition and disease progression, data from the CAPRISA002 study [19], a prospective cohort of HIV seroconverters, was used. In this study, full blood counts were performed at weekly intervals for 3 weeks, fortnightly until 3 months, monthly until 1 year after enrollment, and every 3 months thereafter. Participants who started cART were censored from this analysis.

For analyses of the impact of antituberculous therapy on the ML ratio, data from the CAPRISA003/SAPiT trial were used [23]. In this randomized, controlled trial, participants with HIV infection and tuberculosis received antituberculous therapy followed by cART at one of 3 time points defined by randomization. Full blood counts were performed before randomization and monthly throughout follow-up.

In all cohorts, data were collected in real time during prospective follow-up on standardized case report forms and captured electronically using the DataFax system (Clinical DataFax Systems, Ontario, Canada) by at least 2 data encoders.

### Full Differential Blood Counts

Full blood counts of peripheral blood collected in ethylenediaminetetraacetic acid–containing tubes (Becton Dickinson) were performed by one of 2 clinical diagnostic laboratories, each using a 5-part differential hematology analyzer (Sysmex Model XS, Hamburg, Germany). Full blood counts were subject to strict quality assurance procedures, including twice-daily high and low internal quality control, fortnightly quality controls through the Thistle external quality assurance service (Thistle QA, Johannesburg, South Africa), and annual quality assurance as part of the College of American Pathologists QC scheme. Both laboratories are accredited by the South African National Accreditation System in accordance with international standards ISO 17025:2005 and ISO 15189:2007.

### Statistical Methods

To calculate the ML ratio, the absolute monocyte count was divided by the absolute lymphocyte count. For the primary analysis of tuberculosis incidence, a survival analysis was performed. Study duration was calculated as the time from cART initiation to the first diagnosed episode of tuberculosis or death, withdrawal from the study, transfer to another facility, or last visit date (for patients remaining in follow-up), whichever occurred first. The log-rank test was used to compare survival curves stratified by ML ratio. To test the association between the ML ratio and tuberculosis incidence, a Cox proportional hazards regression model was performed. Covariates included in the model are given in the body text or tables. For the analysis of continuous variables, the Kruskal-Wallis rank sum test was used. All statistical tests were 2-sided, and a *P* value of < .05 was considered statistically significant. Poisson approximations were used to calculate confidence intervals (CIs) for estimations of the incidence rate. Bootstrapped estimates of the adjusted HR across the ML ratio continuum were generated with the boot package. Statistical analyses were performed in R (R Foundation for Statistical Computing), using the following packages: epiR, survival, date, and mfp.

## RESULTS

We assessed whether the ML ratio, when evaluated before commencement of cART, was predictive of the development of tuberculosis during cART in a prospective cohort of 1862 HIV-infected adults in South Africa. Baseline characteristics of this cohort are detailed in Table 1. Notably, the cohort was predominantly female (68.1%) and relatively young (mean age, 24.5 years), and 11.1% of individuals disclosed a previous episode of treated tuberculosis. The median CD4^+^ T-cell count at baseline was 120 cells/µL. The median follow-up for this cohort was 17 months but extended up to 7 years. The incidence of tuberculosis, diagnosed per South African national guidelines, during follow up was 18.79 cases per 1000 patient-years of treatment (95% CI, 14.71–23.66), consistent with comparable cohorts [24, 25].

**Table 1.** Baseline Characteristics of Adults Commencing Combination Antiretroviral Therapy in the Primary Study Cohort, Overall and by Percentile Ranking of the Ratio of Monocytes to Lymphocytes Within the Cohort

Characteristic	Overall (n = 1862)	<5th (n = 93)	5th–95th (n = 1671)	>95th (n = 98)	*P*^a^
Age, y, mean	24.5	24.5	24.6	22.6	.228
Female sex	1268 (68.1)	67 (72.04)	1150 (68.8)	52 (53.1)	.002
Baseline CD4^+^ T-cell count, cells/μL, median (IQR)	120 (61–180)	138 (102–182)	124 (65–182)	36 (17–98)	<.0001
WHO HIV infection/disease stage^b^					
1	355 (19.34)	26 (28.0)	320 (19.2)	9 (9.2)	.004
2	490 (26.69)	22 (23.7)	448 (26.8)	20 (20.4)	.28
3	831 (45.26)	41 (44.1)	739 (44.2)	51 (52.1)	.38
4	160 (8.72)	3 (3.2)	139(8.3)	18 (18.4)	.005
Past history of tuberculosis^c^	206 (11.11)	10 (10.75)	190 (11.37)	6 (6.12)	.27

Data are no. (%) of subjects, unless otherwise indicated.

Abbreviations: HIV, human immunodeficiency virus; IQR, interquartile range; WHO, World Health Organization.

^a^ By the Kruskal-Wallis test.

^b^ A total of 26 patients were missing baseline WHO staging data.

^c^ A total of 7 patients were missing baseline tuberculosis history data.

### ML Ratio Is Associated With Tuberculosis Among Adults Starting cART

Based on Doan and Sabin's observation that extremes of the ML ratio were important for rabbit immunity to *M. bovis* [15], we stratified participants into categories derived from the distribution of the baseline pre-cART ML ratio in the entire cohort. Participants with an ML ratio less than the 5th percentile, between the 5th and 95th percentile, or greater than the 95th percentile were similar in age and the proportion with a previous history of tuberculosis, but males, individuals with lower CD4^+^ T-cell counts, and those with greater WHO staging values were overrepresented in the group with the highest ML ratio (>95th percentile) relative to the other 2 groups. This observation is concordant with expectations of individuals with more severe lymphopenia having higher ML ratios (Table 1).

**Figure 1.**
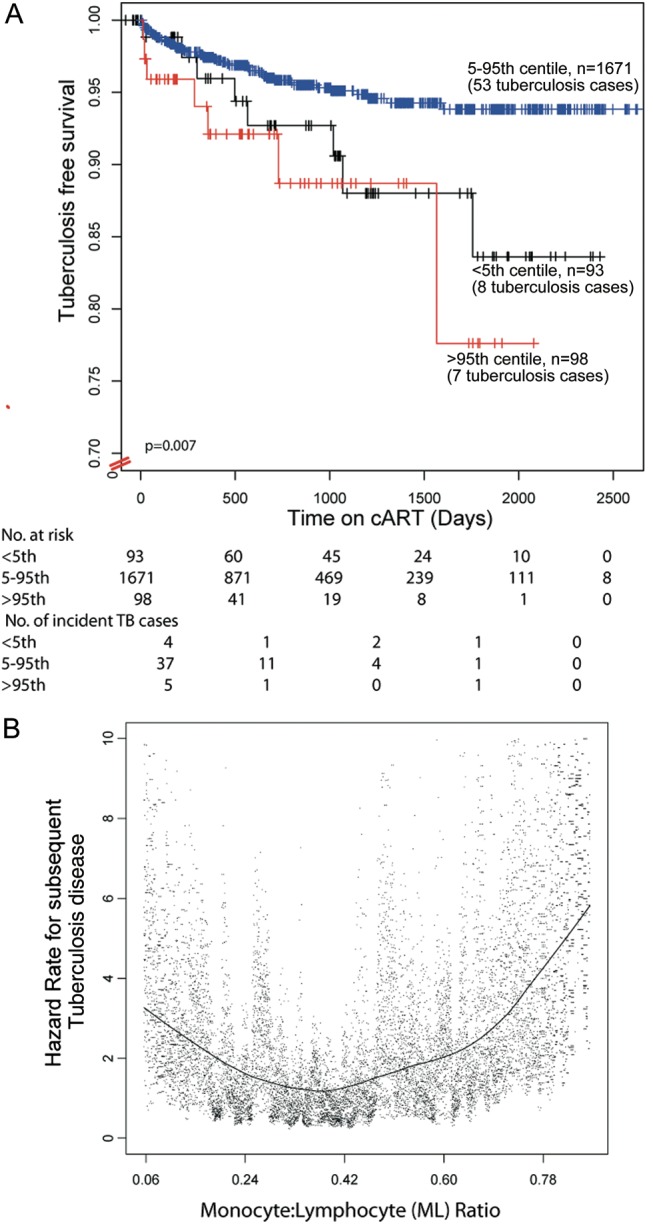
Kaplan-Meier estimates of probability of tuberculosis-free survival for individuals commencing combination antiretroviral therapy (cART), by category of baseline ratio of monocytes to lymphocytes (ML ratio; *A*). Bootstrapped hazard ratio (HR) estimates of tuberculosis across the ML ratio continuum (*B*). HRs are adjusted for age, World Health Organization human immunodeficiency virus infection and disease stage, and past history of tuberculosis. Each dot denotes 1 estimate, with the locally weighted scatterplot smoothing curve overplotted.

The ML ratio before cART initiation was associated with significantly different tuberculosis-free survival probabilities (*P* = .007, by the log-rank test; Figure 1). After exclusion of cases diagnosed within 90 days of cART initiation, which may plausibly represent unmasked tuberculosis, the association remained significant (*P* = .02, by the log-rank test). The overall incidence of tuberculosis among individuals with an ML ratio less than the 5th percentile was 32.61 cases per 1000 patient years (95% CI, 15.38–61.54), and the overall incidence among individuals with an ML ratio greater than the 95th percentile was 51.80 cases per 1000 patient years (95% CI, 23.10–101.71), compared with 16.36 per 1000 patient-years (95% CI, 12.39–21.23) for individuals with an ML ratio between the 5th and 95th percentiles (Table 2). Overall, the incidence of tuberculosis among individuals with an ML ratio less than the 5th percentile or greater than the 95th percentile was 39.44 (95% CI, 23.03–63.39), and the HR was 2.40 (95% CI, 1.35–4.25; *P* = .003). Neither the monocyte count nor lymphocyte count alone were predictive of tuberculosis on formal testing (Supplementary Figure 1 and Table 1).

**Table 2.** Cox Proportional Hazards Modeling of the Tuberculosis Risk Among 1862 Adults, by Percentile Ranking of the Ratio of Monocytes to Lymphocytes Before Initiation of Combination Antiretroviral Therapy

Percentile	Subjects, No.	Subjects Who Developed Tuberculosis, No./Patient-Years	Incidence Rate per 1000 Patient-Years (95% CI)	Unadjusted HR (95% CI)	*P*	Adjusted HR (95% CI)^a^	*P*
**5th–95th**	1671	53/3238.8	16.36 (12.39–21.23)	1.00 (reference)	…	1.00 (reference)	…
<5th (0.1489619)	93	8/245.3	32.61 (15.38–61.54)	1.98 (.945–4.14)	.071	2.09 (.99–4.39)	.053
>95th (0.8746429)	98	7/135.1	51.80 (23.10–101.71)	2.60 (1.19–5.68)	.017	2.63 (1.18–5.83)	.018
<5th or >95th	191	15/380.5	39.44 (23.03–63.39)	2.40 (1.35–4.25)	.003	2.47 (1.39–4.40)	.002

Abbreviations: CI, confidence interval; HR, hazard ratio.

^a^ Adjusted for sex, World Health Organization human immunodeficiency virus infection and disease stage, CD4^+^ T-cell count, and past history of tuberculosis.

To validate these results, the association between the baseline pre-cART ML ratio and the risk of tuberculosis during cART was assessed in a replication cohort of 345 adults commencing cART at an urban treatment site implementing the same care and treatment protocol as in the primary cohort. The overall incidence of tuberculosis after cART commencement in this cohort was 21.62 cases per 1000 patient-years (95% CI, 12.11–35.92). Consistent with the finding in the primary cohort, pretreatment ML ratios that were less than the 5th percentile or greater than the 95th percentile were independently associated with a significantly greater incidence and hazard of tuberculosis (87.68 cases per 1000 patient-years [95% CI, 24.27–233.91] vs 17.64 cases per 1000 patient-years [95% CI, 9.05–31.29]; adjusted HR, 3.94 [95% CI, 1.05–14.73]; *P* = .04).

To refine estimates of the association between the ML ratio and incident tuberculosis, we generated bootstrapped estimates of effect across the ML ratio continuum (Figure 1*B*). These data demonstrate a J-shaped association between ML ratio and incident tuberculosis, with both low and high ML ratios associated with subsequent tuberculosis.

### Association Between ML Ratio and Tuberculosis Risk Is Not Explained by Confounding by Conventional Risk Factors for Tuberculosis

Greater hazards of tuberculosis among those with the highest ML ratios could potentially have been explained by known risk factors for tuberculosis in this population [24, 26] which include a CD4^+^ T-cell of <100 cells/µL, WHO stage 3 or 4 HIV infection or disease, male sex, and/or a past history of tuberculosis. To address these potential confounders, these covariates were included in the Cox proportional hazards model (Table 2). After adjustment for covariates, a pretreatment ML ratio of less than the 5th or greater than the 95th percentile remained independently associated with significantly greater hazards of tuberculosis (adjusted HR, 2.47; 95% CI, 1.39–4.40; *P* = .002). Moreover, the association was consistently observed in analyses stratified for each risk factor individually in the primary cohort (Table 3).

**Table 3.** Stratified Cox Proportional Hazards Modeling of the Tuberculosis Risk Among Individuals With ML Ratios Above the 95th Percentile or Below the 5th Percentile

Factor	Subjects, Proportion^a^	Unadjusted HR (95% CI)	*P*	Adjusted HR (95% CI)^b^	*P*
Sex					
Male	20/571	2.74 (.996–7.55)	.051	2.71 (.966–7.60)	.058
Female	48/1269	2.28 (1.13–4.57)	.021	2.47 (1.22–4.99)	.012
WHO HIV infection/disease stage 3 or 4	59/1656	2.30 (1.25–4.27)	.008	3.18 (1.63–6.21)	.0007
CD4^+^ T-cell count <100 cells/μL	28/656	3.52 (1.59–7.79)	.002	3.36 (1.55–7.69)	.002
Past history of tuberculosis					
Yes	9/206	3.13 (.65–15.13)	.16	3.45 (.70–16.9)	.13
No	56/1656	2.31 (1.25–4.27)	.008	2.40 (1.29–4.45)	.005

Selection of strata was based on previously reported risk factors [24].

Abbreviation: CI, confidence interval; HIV, human immunodeficiency virus; HR, hazard ratio; WHO, World Health Organization.

^a^ Data are no. of subjects who developed tuberculosis/no. at risk.

^b^ Adjusted for sex, WHO HIV infection and disease stage, CD4^+^ T-cell count, and past history of tuberculosis (when that covariate is not the stratification variable).

To exclude the possibility that the effect of ML ratios on the tuberculosis risk was mediated by modification of HIV treatment outcomes, we confirmed that baseline ML ratios were not associated with virological or immunological outcomes (per 2010 WHO criteria) 6 or 12 months after cART initiation in the primary cohort (Supplementary Figure 2) or replication cohort (data not shown).

### ML Ratios Are Stable in HIV-Uninfected Women but Are Increased by HIV Infection and Disease Progression and Normalized by HIV or Tuberculosis Treatment

We assessed how the ML ratio is affected by changes in clinical condition. Intuitively, the ML ratio is expected to vary following HIV acquisition, tuberculosis, cART initiation, or tuberculosis therapy because of changes in lymphocyte counts (Supplementary Figure 1*C* and *D*). Since the index takes into account lymphocyte and monocyte values, and since the latter may be independently regulated, variation of the ML ratio is of interest.

**Figure 2.**
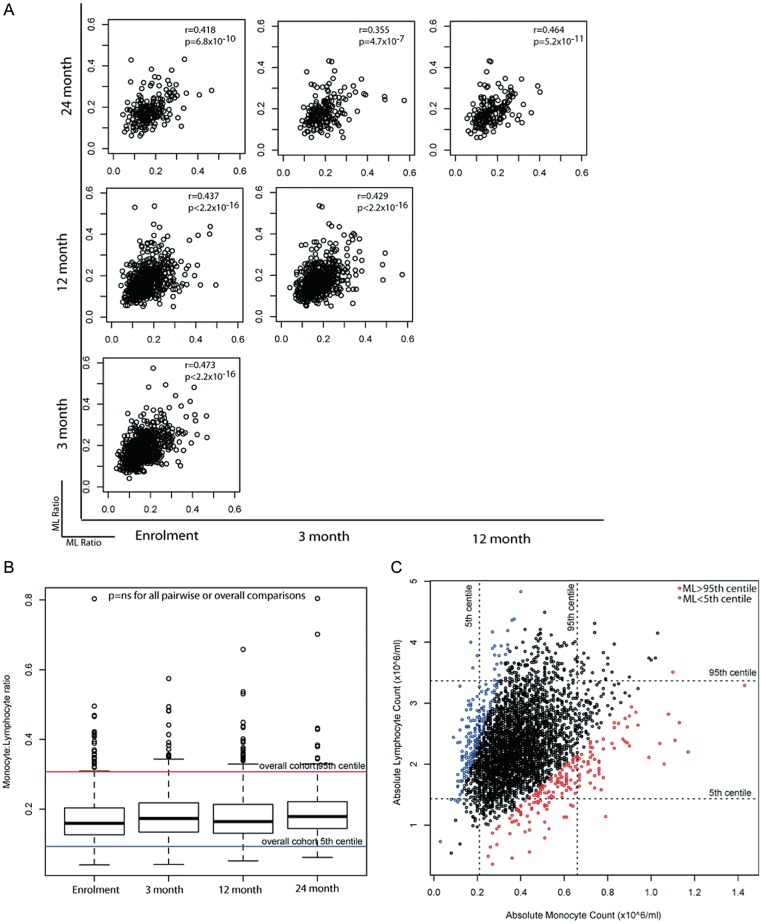
Ratios of monocytes to lymphocytes (ML ratios), monocyte counts, and lymphocyte counts among healthy human immunodeficiency virus–uninfected woman. *A*, The correlation between the ML ratio at enrollment and 3, 12, and 24 months after enrollment among 790 participants. Results of Spearman rank correlation are shown as rho and *P* values in the top right of each correlation plot. *B*, The overall stability of ML ratios in healthy individuals, shown as box plots at each time point. The dotted horizontal lines denote the overall population 5th and 95th percentiles. Boxes show the 25th, 50th (median), and 75th percentiles, whiskers denote the 25th percentile – [1.5 × interquartile range] and the 75th percentile + [1.5 × interquartile range], and outliers are plotted as dots. *C*, ML ratios may be abnormal even if the monocyte or lymphocyte counts are normal. Monocyte and lymphocyte counts are plotted on the *x*-axes and y-axes, respectively. Colors of data points denote the corresponding category of the ML ratio (5th–95th percentiles, >95th percentile, and < 5th percentile). Dotted horizontal lines denote the 5th and 95th percentiles for lymphocyte counts. Dotted vertical lines denote the 5th and 95th percentiles for monocyte counts. Abbreviation: NS, not significant.

The stability of the ML ratio was assessed among 790 healthy, HIV-uninfected South African women who underwent routine safety monitoring over 2 years while enrolled in a topical microbicide gel trial [1]. The ML ratio at visits 0 (enrollment), 3, 12, and 24 months were positively correlated (Spearman rho, 0.35–0.47; *P* < 5 × 10^−7^ for all comparisons; Figure 2*A*), and there was no difference in the distribution of the ML ratio between any 2 visits (Figure 2*B*). Even when monocyte and/or lymphocyte counts were outside of their respective 5th–95th percentiles, the majority (65.7%) of individuals had an ML ratio between the 5th and 95th percentiles (both cell types increased or decreased). Conversely, at 4.3% of visits the monocyte and lymphocyte counts were between the 5th and 95th percentiles, but the ML ratio was not (Figure 2*C*). These data demonstrate that although the ML ratio is stable among healthy individuals, about two thirds of individuals with altered numbers (<5th or >95th percentile) of either cell type retain apparently normal ML ratios, and conversely about 1 in 20 individuals with normal cell numbers may still have altered ML ratios. The ML ratio captures information not adequately captured in the monocyte or lymphocyte counts alone.

**Figure 3.**
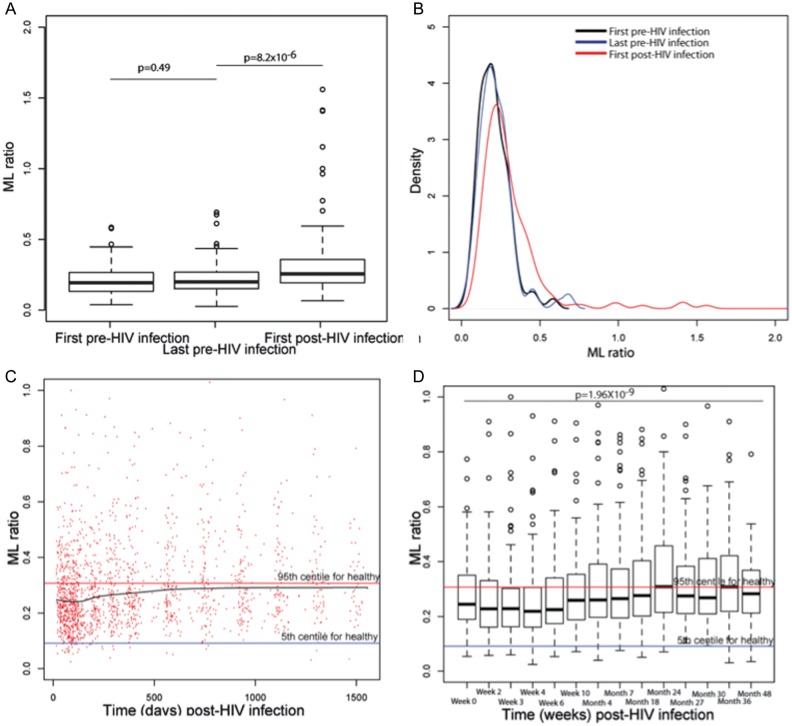
The impact of human immunodeficiency virus (HIV) acquisition and disease progression on ratios of monocytes to lymphocytes (ML ratios). *A*, The ML ratio is stable before infection among 160 women who acquired HIV but is significantly increased by HIV acquisition. Boxes show the 25th, 50th (median), and 75th percentiles, whiskers denote the 25th percentile – [1.5 × interquartile range] and the 75th percentile + [1.5 × interquartile range], and outliers are plotted as dots. *B*, The distribution of ML ratios is broadened by HIV acquisition. *C*, ML ratios over the course of HIV acquisition are variable but increase overall. Data are shown as a scatterplot showing all ML measures over time among HIV acquirers. The black line is a smoothed curve computed by locally weighted scatterplot smoothing. Horizontal lines denote the 5th and 95th percentile for ML ratios in HIV-uninfected healthy individuals in red and blue, respectively. *D*, ML ratios over the course of HIV infection increase significantly with disease progression. Boxes show the 25th, 50th (median), and 75th percentiles, whiskers denote the 25th percentile – [1.5 × interquartile range] and the 75th percentile + [1.5 × interquartile range], and outliers are plotted as dots. Horizontal lines denote the 5th and 95th percentile for ML ratios in HIV-uninfected healthy individuals in red and blue, respectively.

Next, ML ratios were compared before and after HIV acquisition in a cohort of 160 high-risk women prospectively followed for HIV acquisition in several studies [1–3]. ML ratios were similar between enrollment (median, 258 days before infection; interquartile range [IQR], 126–432 days before infection) and the last time point before infection acquisition (median, 97 days before infection; IQR, 43–180 days before infection; Figure 3*A*). In contrast, ML ratios were significantly increased following HIV acquisition (median time of sampling, 29 days after infection; IQR, 15–54 days after infection; median ML ratio, 0.193 before infection vs 0.256 after infection; *P* = 8.2 × 10^−6^; Figure 3*A*), consistent with postinfection lymphopenia. Moreover, the distribution of ML ratios broadened with HIV infection (Figure 3*B*). The ML ratio increased significantly over the course of HIV disease progression (Figure 3*C* and 3*D*; rho for linear correlation with time after infection, 0.17; *P* < 5 × 10^−12^). The ML ratio was not associated with the rate of HIV disease progression, defined as the time to achievement of a CD4^+^ T-cell count of <350 cells/mL, death, or the need for cART (data not shown).

**Figure 4.**
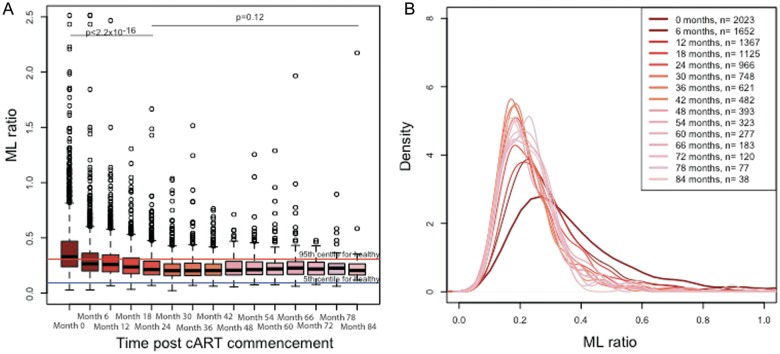
The impact of combination antiretroviral therapy (cART) on the ratio of monocytes to lymphocytes (ML ratio). *A*, The ML ratio is reduced significantly over the first 2 years of cART and stabilizes within the normal range for human immunodeficiency virus (HIV)–uninfected individuals until up to 7 years after cART commencement. Box plots show the ML ratio before and during 7 years of cART. Boxes show the 25th, 50th (median), and 75th percentiles, whiskers denote the 25th percentile – [1.5 × interquartile range] and the 75th percentile + [1.5 × interquartile range], and outliers are plotted as dots. Horizontal lines denote the 5th and 95th percentile for ML ratios in HIV-uninfected healthy individuals in red and blue, respectively. *B*, The distribution of ML ratios becomes narrower over time.

The change in ML ratio with duration of cART was assessed in a cohort of 2023 individuals who underwent screening and commenced cART. The ML ratio was significantly altered by cART: over the first 24 months of therapy, the median ML ratio decreased to within the range observed for HIV-uninfected healthy volunteers and was maintained at this level for up to 7 years (Figure 4).

Finally, the impact of tuberculosis therapy on the ML ratio was evaluated. Among 642 adults with tuberculosis and HIV infection who commenced antituberculosis therapy before cART [4], antituberculosis therapy significantly reduced ML ratios (median ML ratio, 0.373 [IQR, 0.273–0.53] before treatment vs 0.2753 [IQR, 0.203–0.376] after treatment; *P* = 3.32X10^−14^). Before tuberculosis therapy, 15% of individuals (96/642) had ML ratios in the normal range for an HIV-uninfected population, compared with 41.7% (189/642; median therapy duration, 9 days) following tuberculosis therapy (*P* < .0001).

## DISCUSSION

The ability to stratify patients starting cART on the basis of their risk of tuberculosis during follow-up is necessary for targeting prevention interventions. Here, we demonstrate that extremes in the ratio of peripheral blood monocytes to lymphocytes, but neither monocyte or lymphocyte counts alone, were associated with an increased risk of incident tuberculosis over several years. Our observation that the ML ratio is associated with tuberculosis risk supports findings of recent microarray studies in HIV-negative infants (Fletcher et al, unpublished data) and extends findings made >80 years ago in rabbit models of mycobacterial infection [15]. In more recent studies in cattle, the ratio of monocyte-derived macrophages to lymphocytes has been shown to correlate with inhibition of mycobacterial growth in vitro [27, 28]. The observation that risk is higher among individuals with either low or high ML ratios adds evidence to support recent findings that extremes of immunity are associated with tuberculosis [29, 30], as differences in the ratio of myeloid transcripts to lymphoid transcripts in infants were associated with differences in inflammatory gene expression (Fletcher et al, unpublished data). Collectively, the historical small-animal studies, recent microarray data, and these human studies demonstrate that the ML ratio may be an easily measured predictive biomarker of tuberculosis. This ratio could herald a previously unknown pathophysiologic driver of tuberculosis.

Whether the ML ratio is causatively involved in tuberculosis or simply reflects underlying traits that affect tuberculosis risk will require further study. It is likely that the ML ratio captures underlying differences in the immune microenvironment, as has been implied by its prognostic value in influenza [31] and malaria. Recent studies in mice [32] and humans [33] have shown that subsets of hematopoietic stem cells have distinct biases in the ratio of myeloid and lymphoid cells they give rise to [34, 35]. We speculate that differences in the proportions of myeloid-biased, balanced, or lymphoid-biased hematopoietic stem cells may underlie the peripheral differences in the ML ratio and that the ontogeny of monocytes and lymphocytes, reflected in their ratio, may affect their ability to respond to mycobacterial infection. Recent studies have demonstrated that mycobacterial infection may alter hematopoiesis [36] or directly infect bone marrow mesenchymal stem cells [37]. Therefore, it is plausible that latent or prior *M. tuberculosis* infection may alter hematopoietic stem cells such that the peripheral ML ratio is altered. In support of this is the observation that in the microarray studies, alongside the ratio of myeloid transcripts to lymphoid transcripts being a signature of risk, transcripts suggesting activation/quiescence of hematopoietic stem cells were more prevalent among infants who developed tuberculosis. Alternatively, the ML ratio and underlying hematopoietic stem cell activity may be epiphenomena, and the ML ratio may be a marker of where along the spectrum [38] of tuberculosis between latent and active disease an individual is, consistent with hypothesis first proposed in 1935 by Kato [39]. Further work to explore the pathophysiological involvement of the ML ratio in tuberculosis may yield new pathways to modify or prevent disease.

The clinical usefulness of the observation that the ML ratio is related to prospective risk of tuberculosis is promising but will benefit from extension and validation in non–tuberculosis-endemic regions, among children, and in HIV-negative adults. In particular, the diagnostic performance of the ratio and the development of standards or diagnostic thresholds require attention. Evaluation of whether the ML ratio is associated with other disorders, as is suggested by recent studies in influenza [31] and malaria [40], is also necessary. Moreover, the question of whether ML ratios during cART continue to offer predictive value will need specific evaluation. Since neither the ML ratio, TST, nor interferon γ–release assays reliably detect all individuals at risk for tuberculosis, their combined use may need to be explored for its potential in risk stratification.

Although we observed that higher ML ratios were associated with tuberculosis, it is possible that tuberculosis was present but not diagnosed at the time of enrollment (reverse causation). But the observation that the association appears to hold out for several years, and is present even excluding early tuberculosis diagnoses, suggests that undiagnosed tuberculosis at enrolment does not account for the association.

An important potential limitation of this study is that this was a secondary data analysis study, so it is plausible that there may have been misclassification of the tuberculosis diagnosis if tuberculosis was not specifically and prospectively assessed. But the data used here were obtained during the conduct of prospective studies in which assessment for any clinical disorder, particularly tuberculosis, was performed. The diagnostic algorithm for tuberculosis used in these studies followed the national South African guidelines, and so the diagnostic procedures in this study approximate those in clinical settings, which are known to suffer impaired sensitivity for incident tuberculosis. A further limitation of this study is that participants did not have TSTs or interferon γ–release assays performed. Finally, at the time of clinical assessment, although clinicians were not blinded to the full blood count, they were not aware of this hypothesis, thus diminishing diagnostic bias.

Among patients starting cART, the ability to predict the risk of tuberculosis may facilitate targeted interventions such as vaccination, treatment for latent *M. tuberculosis* infection to prevent tuberculosis ,or closer clinical monitoring to diagnose tuberculosis earlier. The ML ratio may be a useful, readily available tool to stratify risk of tuberculosis and provide a new insight into tuberculosis pathogenesis.

## Supplementary Data

Supplementary materials are available at *The Journal of Infectious Diseases* online (http://jid.oxfordjournals.org/). Supplementary materials consist of data provided by the author that are published to benefit the reader. The posted materials are not copyedited. The contents of all supplementary data are the sole responsibility of the authors. Questions or messages regarding errors should be addressed to the author.

Supplementary Data
