# Supplementary material for: Ratio of Monocytes to Lymphocytes in Peripheral Blood Identifies Adults at Risk of Incident Tuberculosis Among HIV-Infected Adults Initiating Antiretroviral Therapy
Source: J Infect Dis. 2013 Sep 16;209(4):500–9. doi: 10.1093/infdis/jit494 (PMC3903371; doi:10.1093/infdis/jit494)
Supplement: Supplementary Data [file supp_209_4_500__index.html]

Blood monocyte–lymphocyte ratios identify adults at risk of incident tuberculosis amongst patients initiating antiretroviral therapy — Ratio of Monocytes to Lymphocytes in Peripheral Blood Identifies Adults at Risk of Incident Tuberculosis Among HIV-Infected Adults Initiating Antiretroviral Therapy — Ratio of Monocytes to Lymphocytes in Peripheral Blood Identifies Adults at Risk of Incident Tuberculosis Among HIV-Infected Adults Initiating Antiretroviral Therapy — Supplementary Data 

# Ratio of Monocytes to Lymphocytes in Peripheral Blood Identifies Adults at Risk of Incident Tuberculosis Among HIV-Infected Adults Initiating Antiretroviral Therapy

## Supplementary Data

Supplementary Data

**Files in this Data Supplement:**

- Supplementary Data - Docx file
- Supplementary Figure 1 - tif file
- Supplementary Figure 2 - png file
