# Supplementary material for: Ratio of Monocytes to Lymphocytes in Peripheral Blood Identifies Adults at Risk of Incident Tuberculosis Among HIV-Infected Adults Initiating Antiretroviral Therapy
Source: J Infect Dis. 2013 Sep 16;209(4):500–9. doi: 10.1093/infdis/jit494 (PMC3903371; doi:10.1093/infdis/jit494)
Supplement: Supplementary Data [file supp_jit494_jit494supp.docx]

**Supplementary Appendix**

Blood monocyte – lymphocyte ratios identify adults at risk of incident tuberculosis among adults initiating antiretroviral therapy

**Contents**

Supplementary Figures 2

Figure S1 2

Figure S2 3

Supplementary Tables 4

Table S1 4

#

# Supplementary Figures

## Figure S1

Kaplan-Meier estimates of probability of TB-disease free survival for individuals commencing cART, by centile category of baseline monocyte **(Panel A)** or lymphocyte **(Panel B)** count showing that neither the monocyte count nor the lymphocyte count are associated with TB-disease free survival for individuals commencing cART. Both the monocyte count (**Panel C**) and the lymphocyte count (**Panel D**) contribute to the Monocyte:Lymphocyte (ML) ratio.

## Figure S2

ML ratios at baseline (pre-cART commencement) are similar regardless of whether patients respond by **(Panel A)** 6 months to cART with virological suppression (<5000 copies/ml) and immunological reconstitution (a 50 cells/μl increase from baseline) (V+/I+), virological suppression without immunological reconstitution (V+/I-), failure to suppress viraemia but successful immunological reconstitution (V-/I+) or failure to suppress viraemia and failure to reconstitute CD4+ T-cell counts (V-/I-). Box plots show baseline ML ratios for each category of treatment responders. ML ratios at baseline (pre-cART commencement) are similar regardless of whether patients respond to cART by 12 months. **(Panel B)** Data shown as above but with outcomes at 12 months defined as virological suppression (<5000 copies/ml) and immunological reconstitution (a 100 cells/μl increase from baseline).

# **Supplementary Tables**

## Table S1

Cox-proportional hazards modeling of tuberculosis risk by pre-cART monocyte or lymphocyte category, n=1862.

| Explanatory Variable | N | No. of patients developed TB/patient-years | Incidence Rate per 1000 patient years(95% CI) | Unadjusted HR (95% CI) | p-value | *Adjusted HR (95% CI) | p-value |
| --- | --- | --- | --- | --- | --- | --- | --- |
| **Monocyte count 5^th^-95^th^ percentile for patients commencing cART** | 1685 | 65/3301·04 | 19·69 (15·33-24·93) | 1·00 (REFERENCE) | - | 1·00  (REFERENCE) | - |
| Monocyte count <5^th^ percentile for patients commencing cART | 81 | 1/186·4 | 5·37 ( 0·49-25·02) | 0·31 (0·04-2·21) | 0·24 | 0·30 (0·04-2·18) | 0·24 |
| Monocyte count >95^th^ percentile for patients commencing cART | 96 | 2/179·23 | 15·16 (3·02-48·61) | 0·68 (0·17-2·79) | 0·60 | 0·65 (0·15-2·65) | 0·54 |
| **Lymphocyte count 5^th^-95^th^ percentile for patients commencing cART** | 1672 | 61/3291·4 | 18·53 (14·31-23·64) | 1·00  (REFERENCE) | - | 1·00  (REFERENCE) | - |
| Lymphocyte count <5^th^ percentile for patients commencing cART | 96 | 4/164·73 | 24·28 (8·12-57·73) | 1·28 (0·47-3·52) | 0·633 | 1·32 (0·47-3·69) | 0·60 |
| Lymphocyte count >95^th^ percentile for patients commencing cART | 94 | 3/163·2 | 18·38 (5·08-49·04) | 0·94 (0·30-3·0) | 0·92 | 0·90 (0·28-2·89) | 0·85 |

*adjusted for sex, WHO stage, CD4 count, past history of TB
