# Supplementary figures and images for: Ratio of Monocytes to Lymphocytes in Peripheral Blood Identifies Adults at Risk of Incident Tuberculosis Among HIV-Infected Adults Initiating Antiretroviral Therapy
Source: J Infect Dis. 2013 Sep 16;209(4):500–9. doi: 10.1093/infdis/jit494 (PMC3903371; doi:10.1093/infdis/jit494)

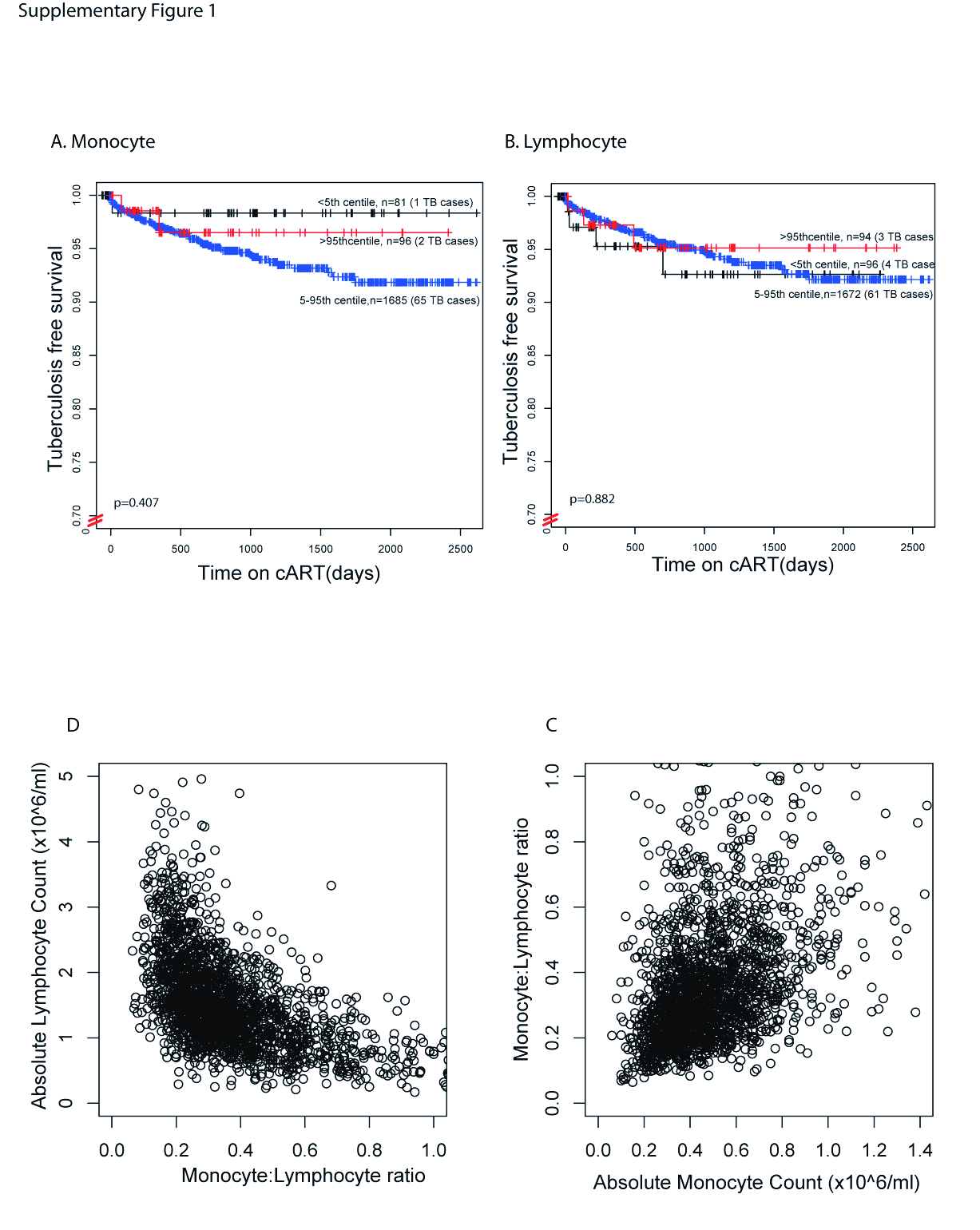

Supplement: Supplementary Data [file supp_jit494_jit494supp_fig1.tif]

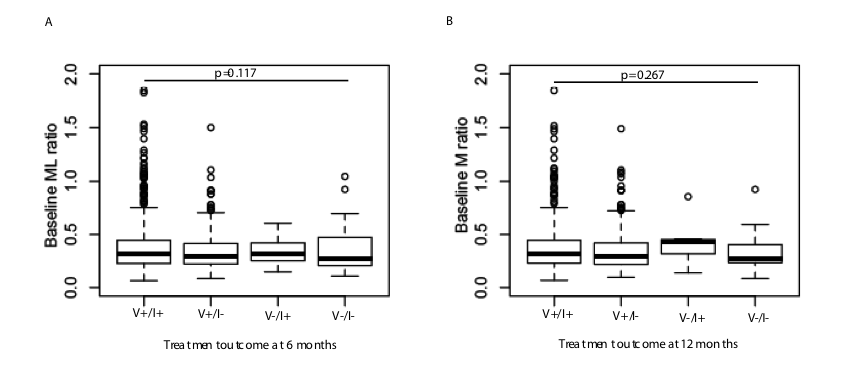

Supplement: Supplementary Data [file supp_jit494_jit494supp_fig2.png]
